# Spatiotemporal distribution of migraine in China: analyses based on baidu index

**DOI:** 10.1186/s12889-023-16909-9

**Published:** 2023-10-10

**Authors:** Liling Lin, Mengyi Zhu, Junxiong Qiu, Qiang Li, Junmeng Zheng, Yanni Fu, Jianwei Lin

**Affiliations:** 1 Department of Anesthesiology, Sun Yat-sen Memorial Hospital, Sun Yat-sen University, Guangzhou, China; 2 Zhongshan School of Medicine, Sun Yat-sen University, Guangzhou, China; 3 Department of Cardiovascular Surgery, Sun Yat-sen Memorial Hospital, Sun Yat-sen University, Guangzhou, China; 4 Department of Anesthesiology, Sun Yat-sen University Cancer Center, Sun Yat-sen University, Guangzhou, China; 5 Big Data Laboratory, Joint Shantou International Eye Center of Shantou University and The Chinese University of Hong Kong, Shantou, Guangdong China; 6 Big Data AI Laboratory, Shenshan Medical Center, Sun Yat-sen Memorial Hospital, Sun Yat-sen University, Shanwei, Guangdong China

**Keywords:** Migraine, Baidu index, Prevalence, Spatiotemporal distribution, Infodemiology

## Abstract

**Background:**

In recent years, innovative approaches utilizing Internet data have emerged in the field of syndromic surveillance. These novel methods aim to aid in the early prediction of epidemics across various scenarios and diseases. It has been observed that these systems demonstrate remarkable accuracy in monitoring outbreaks even before they become apparent in the general population. Therefore, they serve as valuable complementary tools to augment existing methodologies. In this study, we aimed to investigate the spatiotemporal distribution of migraine in China by leveraging Baidu Index (BI) data.

**Methods:**

Migraine-related BI data from January 2014 to December 2022 were leveraged, covering 301 city-level areas from 31 provincial-level regions by using the keyword “migraine (偏头痛)”. Prevalence data from the Global Burden of Disease study (GBD) were attracted to ensure the reliability of utilizing migraine-related BI data for research. Comprehensive analytical methods were then followed to investigate migraine’s spatiotemporal distribution. The Seasonal-Trend decomposition procedure based on Loess (STL) was used to identify the temporal distribution. Spatial distribution was explored using the Getis-Ord Gi^*^ statistic, standard deviation ellipse analysis, Moran’s Index, and Ordinary Kriging. The top eight migraine-related search terms were analyzed through the Demand Graph feature in the Baidu Index platform to understand the public’s concerns related to migraine.

**Results:**

A strong association was observed between migraine-related BI and the prevalence data of migraine from GBD with a Spearman correlation coefficient of 0.983 (*P* = 4.96 × 10^− 5^). The overall trend of migraine-related BI showed a gradual upward trend over the years with a sharp increase from 2017 to 2019. Seasonality was observed and the peak period occurred in spring nationwide. The middle-lower reaches of the Yangtze River were found to be hotspots, while the eastern coastal areas had the highest concentration of migraine-related BI, with a gradual decrease towards the west. The most common search term related to migraine was “How to treat migraine quickly and effectively (偏头痛怎么办最快最有效的方法)”.

**Conclusions:**

This study reveals important findings on migraine distribution in China, underscoring the urgent need for effective prevention and management strategies.

**Supplementary Information:**

The online version contains supplementary material available at 10.1186/s12889-023-16909-9.

## Background

Migraine is a prevalent neurological disorder affecting about 14% of the global population, characterized by recurring headaches, accompanied by nausea, vomiting, and sensitivity to light and sound [1]. As migraine is a type of headache disorder, it ranks second in years lived with disability (YLDs) worldwide [2]. It is worth noting that East Asia, particularly China, has witnessed a remarkable surge in migraine incidence rates. In 2019, China reported an astonishing 188.93 million migraine cases as measured by prevalence, illustrating the significant burden this disorder places on the nation [1, 2]. Despite its significant impact on individuals and society, the exact factors contributing to the occurrence of migraine remain unclear, particularly in the context of its spatiotemporal distribution.

Spatiotemporal analysis, a multidisciplinary approach that examines the distribution of diseases, events, or phenomena across both geographical space and time, has emerged as a powerful tool in the field of public health and healthcare management [3]. This analytical framework facilitates a deeper understanding of the complex interplay between geographic locations, temporal trends, and health-related outcomes. By considering the spatial and temporal dimensions together, researchers and policymakers can uncover insightful patterns, identify influential factors, and develop targeted strategies to enhance healthcare provision, disease prevention, and resource allocation [4, 5].

To develop effective strategies, understanding the spatiotemporal distribution of migraine is essential. However, traditional methods such as surveys and medical records are time-consuming and expensive. In contrast, the Internet has emerged as a cost-effective alternative to traditional research methods. Several studies have used search engine data like Google Trends to explore spatiotemporal patterns of health conditions such as meningitis, measles, and respiratory syncytial virus infections [6–8]. Google Trends is a popular tool for tracking search interests worldwide, but it is not accessible in Mainland China. As of March 2023, China had surpassed 1 billion internet users, with over 90% of web surfers in China preferring Baidu, which dominates China’s search engine market, akin to Google in Western countries [9]. However, to the best of our knowledge, no study has used Baidu Index (BI) data to explore the spatiotemporal distribution of migraine in China. Notably, migraine sufferers often self-medicate [10], but inappropriate self-treatment can lead to severe social and health issues [11–13]. Therefore, there is a pressing need to uncover the search behavior by using BI data.

By utilizing BI data, this study aims to address three fundamental aspects regarding migraine in China: (i) unveiling the seasonal trends for migraine; (ii) investigating the spatial distribution for migraine; and (iii) identifying dominant search needs associated with migraine. This study analyzes the spatiotemporal distribution and characteristics of migraine in China and aims to offer epidemiological insights, guiding public health policies and interventions to alleviate the migraine burden. Additionally, it introduces a novel approach to leveraging internet data for exploring health-related concerns.

## Methods

The Baidu Index (BI, https://index.baidu.com/v2/index.html#/) was used to investigate the search behavior regarding migraine in Mainland China. Baidu Index is a data-sharing platform leveraging comprehensive user behavioral data. It quantifies the weighted frequencies of unique keyword searches, reflecting keyword popularity relative to Baidu’s total search volume [9, 14]. The creation of BI involves intricate data refinement and weighting procedures conducted by IT experts, accounting for keyword variations and derivations [15]. The search term “migraine (偏头痛)” was chosen to explore the search behavior, due to its common recognition as a term for headache and its availability in BI. Migraine-related BI data were obtained over a 9-year period, from January 1, 2014, to December 31, 2022, covering a total of 301 cities at the city level, located within 31 provinces, municipalities, and autonomous regions at the province level. The data collected included daily, weekly, monthly, and yearly averages, providing a detailed understanding of migraine-related search behaviors.

### Correlation between trends in the Baidu Index and the prevalence of migraine

The interpretation and reliability of the results can be incorrect if the study solely depends on Internet search data to explore health condition patterns without real-world validation. Thus, we utilized migraine prevalence data from the Global Burden of Disease (GBD) study (available at https://vizhub.healthdata.org/gbd-results/) from 2011 to 2019. Subsequently, a correlation analysis was performed between the GBD prevalence data and the BI data to establish the validity of the latter as a reliable metric. The GBD is an authoritative source that provides prevalence data for various diseases across countries and regions [2].

### Trend strength and seasonal strength of BI in Mainland China about migraine

The Simple exponential smoothing method with the k value of 7 was applied to smooth the time series data [16]. The multiplicative model is used for further analysis as the BI time series data is dependent on the year after applying the Simple exponential smoothing method [17]. The Seasonal-Trend decomposition procedure based on Loess (STL) was used to separate the time series into trend, seasonal, and residual components [18].

### Spatial distribution of BI about migraine

The spatial distribution of BI related to migraine across various provinces in China was explored using the Getis-Ord Gi^*^ statistic and standard deviation ellipse analysis [19], for its advantage in identifying hotspots and clusters of migraine. The standard deviation ellipse was used to provide a graphical representation of the direction for spatial distribution of migraine. The spatial autocorrelation of migraine-related BI was analyzed using Moran’s Index [20]. Global Moran’s I was used to identify overall clustering or dispersion patterns by assessing the spatial autocorrelation among the BI values. Local Moran’s I was applied for a more detailed examination of spatial heterogeneity and to identify specific locations of clustering or dispersion through local autocorrelation analysis [21]. The Ordinary Kriging, which utilizes spatial autocorrelation to estimate values at unsampled locations, was applied as an additional method to further visualize the spatial distribution of the migraine [22].

### Migraine-related demand graphs

To offer a deeper understanding of the public’s information-seeking behavior and provide a foundation for evidence-based strategies to improve migraine awareness and management, we employed a complementary approach to further enrich our investigation into “migraine (偏头痛)”. Utilizing the built-in feature of Baidu Index, known as the Demand Graph, we collected data on the top eight demand terms related to “migraine (偏头痛)”. According to the official documentation of Baidu Index (https://index.baidu.com/v2/main/index.html#/help), the Demand Graph feature can assist researchers in identifying central keywords and pinpointing pain points within information needs and product services among internet users. This step was taken to explore search demands associated with migraine across different seasons [23].

### Statistical analysis

The Spearman correlation analysis was applied to explore the association between migraine-related BI data and prevalence of migraine. A *P* value less than 0.05 (two-sided) was deemed as significant.

The temporal pattern of migraine was analyzed using the STL method. Prior to that, a log transformation was applied to the original data to mitigate the impact of extreme values and stabilize the variance. The trend and seasonal strength for migraine was obtained based on time series BI data, using the following formulas [24]:

$$\begin{array}{l}{\rm{for\, trend\, strength}}: {{\rm{F}}_{\rm{T}}} = {\rm{max}}\left({0,\,1 - \frac{{{\rm{Var}}({{\rm R}_{\rm{t}}})}}{{{\rm{Var}}({{\rm T}_{\rm{t}}} + {{\rm R}_{\rm{t}}})}}} \right),{\rm{and}}\end{array}$$

$$\begin{array}{l}{\rm{for\, seasonal\, strength}}:{{\rm{F}}_{\rm{S}}} = {\rm{max}}\left({0,\,1 - \frac{{{\rm{Var}}({{\rm R}_{\rm{t}}})}}{{{\rm{Var}}({{\rm{S}}_{\rm{t}}} + {{\rm R}_{\rm{t}}})}}} \right),\end{array}$$

where F_T_ is the trend strength, F_S_ is the seasonal strength, R_t_ is the remainder component, T_t_ is the trend component, S_t_ is the seasonal component. A seasonal strength value below 0.4 was considered indicative of the absence of a seasonal trend [15].

To construct the Ordinary Kriging model, data from 301 city-level sources were utilized. Prior to that, measures such as data cleaning, outlier detection, and missing value imputation were undertaken to ensure the validity of the results [25]. The normal distribution, semivariogram, and second-order trend analysis were conducted on the migraine-related BI data to provide evidence supporting the suitability of Ordinary Kriging interpolation [26, 27].

All analyses and visualization were carried out using the ArcGIS 10.7 software and the following packages: forecast (v8.2.1), ggplot2 (v3.4.2), and plotrix (v3.8-2) in R (v4.1.3).

## Results

**Fig. 1:**
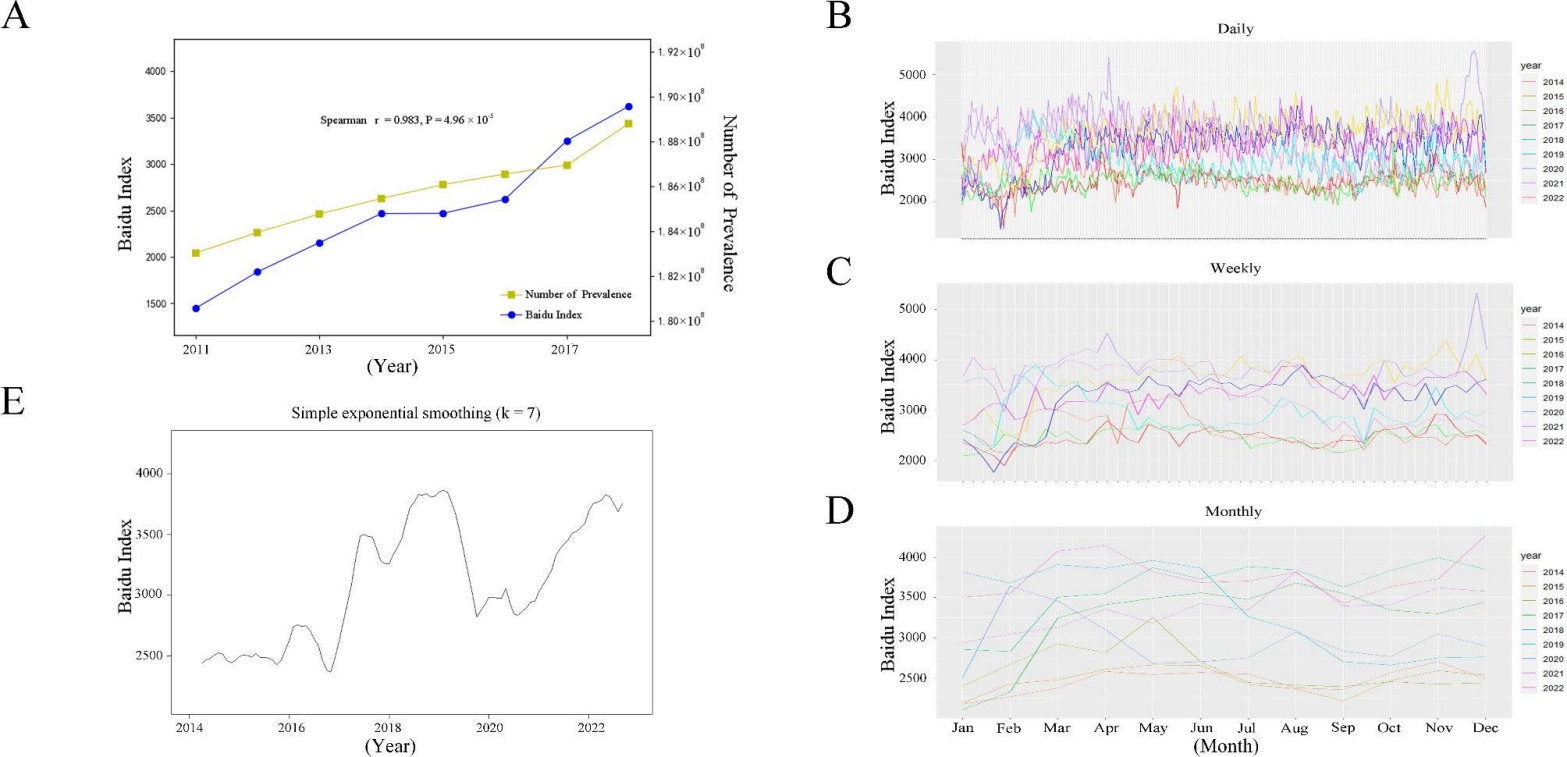
Trends and seasonality of migraine-related BI data in Mainland China. **(A)** Spearman correlation between migraine-related BI data and the prevalence of migraine; **(B)** Overall trends by daily BI data across different years; **(C)** Overall trends by weekly BI data across different years; **(D)** Overall trends by monthly BI data across different years; **(E)** Smoothing time-series curve by using the Simple exponential smoothing method. BI, Baidu Index

Based on the analysis of the migraine-related BI data and the prevalence of migraine, a strong positive correlation was found between the two datasets (Spearman correlation coefficient r = 0.983, *P* = 4.96 × 10^− 5^) as shown in Fig. 1A, suggesting that there is a significant relationship between the two datasets, which also supports the potential use of BI data as a reliable source for monitoring the spatiotemporal distribution of migraine in China.

### Seasonal variation of migraine in Mainland China

**Fig. 2:**
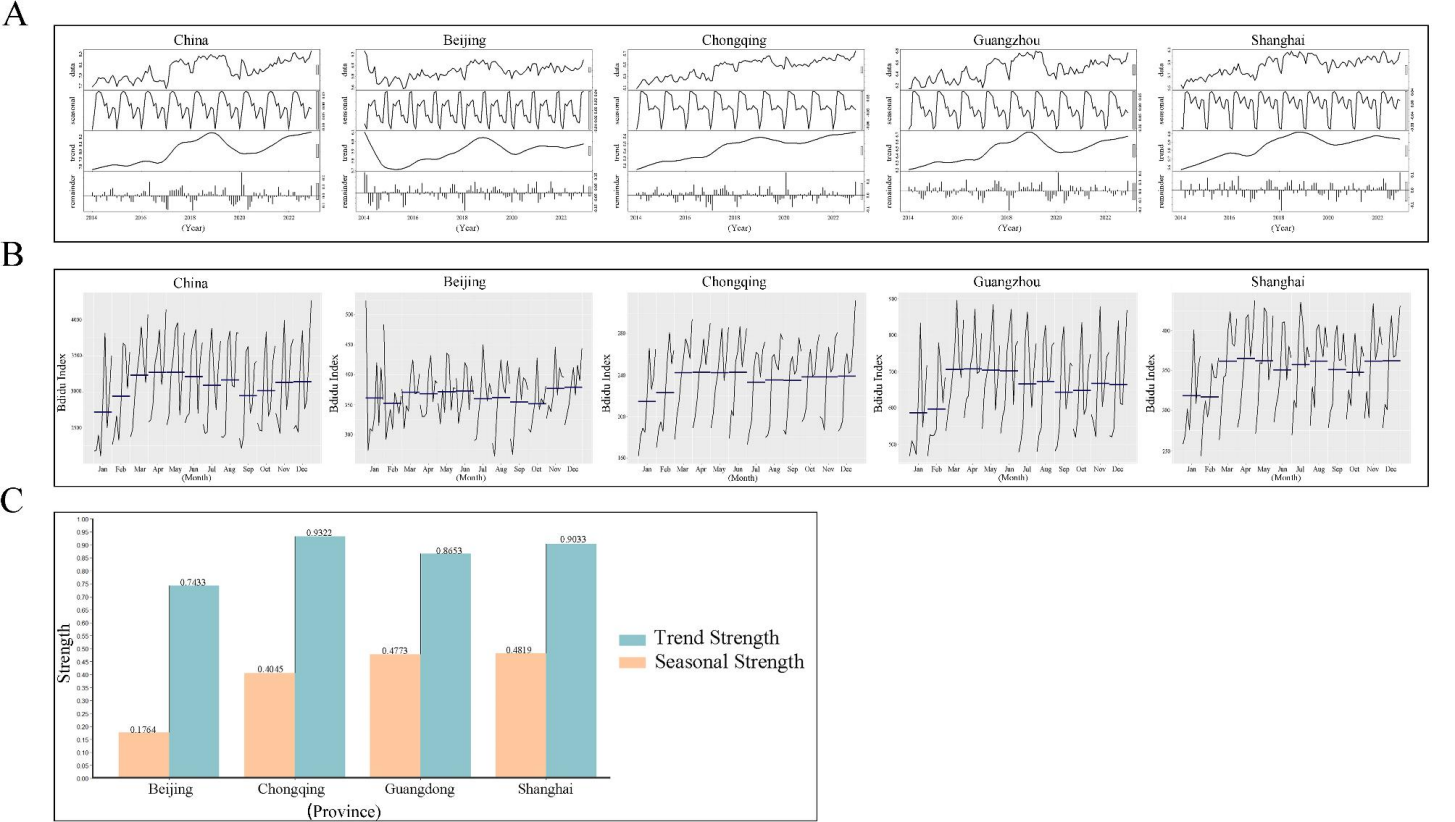
Trends and seasonality of migraine in Mainland China based on migraine-related Baidu Index data. **(A)** Decomposition time-series curve by the Seasonal-Trend decomposition procedure based on Loess method for Mainland China and four province-level regions; **(B)** Subseries seasonal plot for Mainland China and four province-level regions; **(C)** Trend strength and seasonal strength of migraine for four province-level regions

The overall trend of the search for migraine showed a gradual upward trend over the years. There was a sharp increase in BI from 2017 to 2019, followed by a more gradual increase, as shown in Fig. 1B and E. The STL analysis also showed that the overall trend was increasing, with the seasonal component showing fluctuations around 0, indicating the presence of seasonality in the search for migraine (Fig. 2A). The analysis of the residual component using kernel density estimation showed an approximately normal distribution, demonstrating the validity of using STL to decompose the BI data (Figure S1). The results from the seasonal subplots showed that, the peak period occurred in March, April, and May each year nationwide (Fig. 2B). Four specific representative regions that have similar levels of economic development, population density, and Internet penetration, including Beijing, Shanghai, Guangdong, and Chongqing were selected to represent the northern, eastern, southern, and western regions of China. The seasonal subplots for these four regions also showed that the peak period occurred in March, April, and May each year (Fig. 2B). Figure 2 C demonstrates the overall trend and seasonal strength in the four regions. Shanghai displayed the highest seasonal strength (0.481), followed by Guangdong and Chongqing. Conversely, Beijing exhibited the weakest seasonal strength, with a value of 0.176. In terms of trend strength, Chongqing showed the strongest with the trend strength of 0.932, while Beijing exhibited the lowest level of seasonal strength compared to the other three regions.

### Spatial variation of migraine in Mainland China

**Fig. 3:**
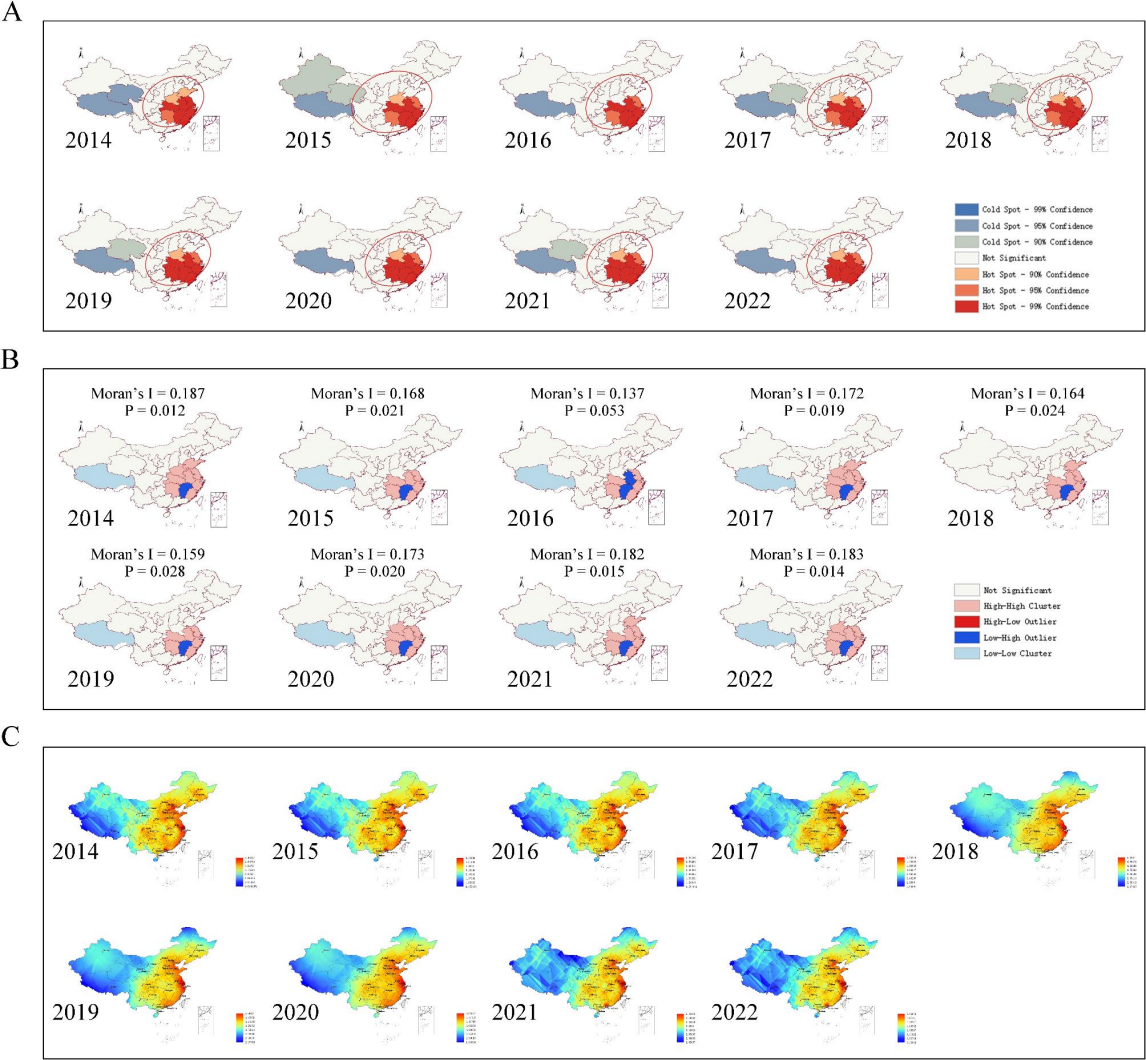
Spatial distribution of migraine in Mainland China based on migraine-related BI data. **(A)** Hotspot analysis and directional distribution of migraine in Mainland China by using 31 province-level Baidu Index data; **(B)** Global (Moran’s I index) and local spatial autocorrelation in Mainland China by using 31 province-level BI data; **(C)** Ordinary Kriging interpolation of migraine in Mainland China by using 301 city-level BI data. BI, Baidu Index

The Getis-Ord Gi^*^ statistic identified hotspots and clusters for migraine, which were found to be mainly concentrated in the eastern region of China, particularly in the middle and lower reaches of the Yangtze River. Conversely, cold spots were found to be mainly in the western region of China. The standard deviation ellipse analysis showed that the distribution of migraine was relatively stable over time, gradually weakening from east to west (Fig. 3A). The results from Global Moran’s Index showed that all years had a global Moran’s I value greater than 0 and a P-value less than 0.05, except for 2016 (global Moran’s I = 0.137, *P* = 0.053), indicating the presence of clustering in the data (Fig. 3B). Meanwhile, the findings from the Local Moran’s I showed that the High/High cluster was mainly concentrated in the eastern region of China while the Low/Low cluster was mainly in the western. It is worth noting that Jiangxi Province, located in eastern region, was a significant Low-High Outlier (Fig. 3B).

Figure S2A-B depicts the distribution of migraine-related BI data, which approximates a normal distribution. Meanwhile, Figure S2C demonstrates the presence of spatial dependence through the semivariogram function, indicating the suitability of employing Kriging techniques [26]. After conducting second-order trend analysis and observing a discernible trend (Figure S2D), we proceeded to eliminate the second-order trend prior to carrying out Ordinary Kriging interpolation. Figure 3 C illustrates the spatial distribution of migraine-related BI based on the Ordinary Kriging method. The results indicate that the eastern coastal regions exhibit the highest concentration of migraine-related BI values, gradually decreasing towards the west. Shanghai, Zhejiang, and Jiangsu provinces show the highest BI values, while the western and northwestern regions, including Tibet, Qinghai, and Xinjiang, exhibit the lowest values. These findings highlight a clear regional disparity in the spatial distribution of migraine-related BI in Mainland China.

### Migraine-related demand graphs

**Fig. 4:**
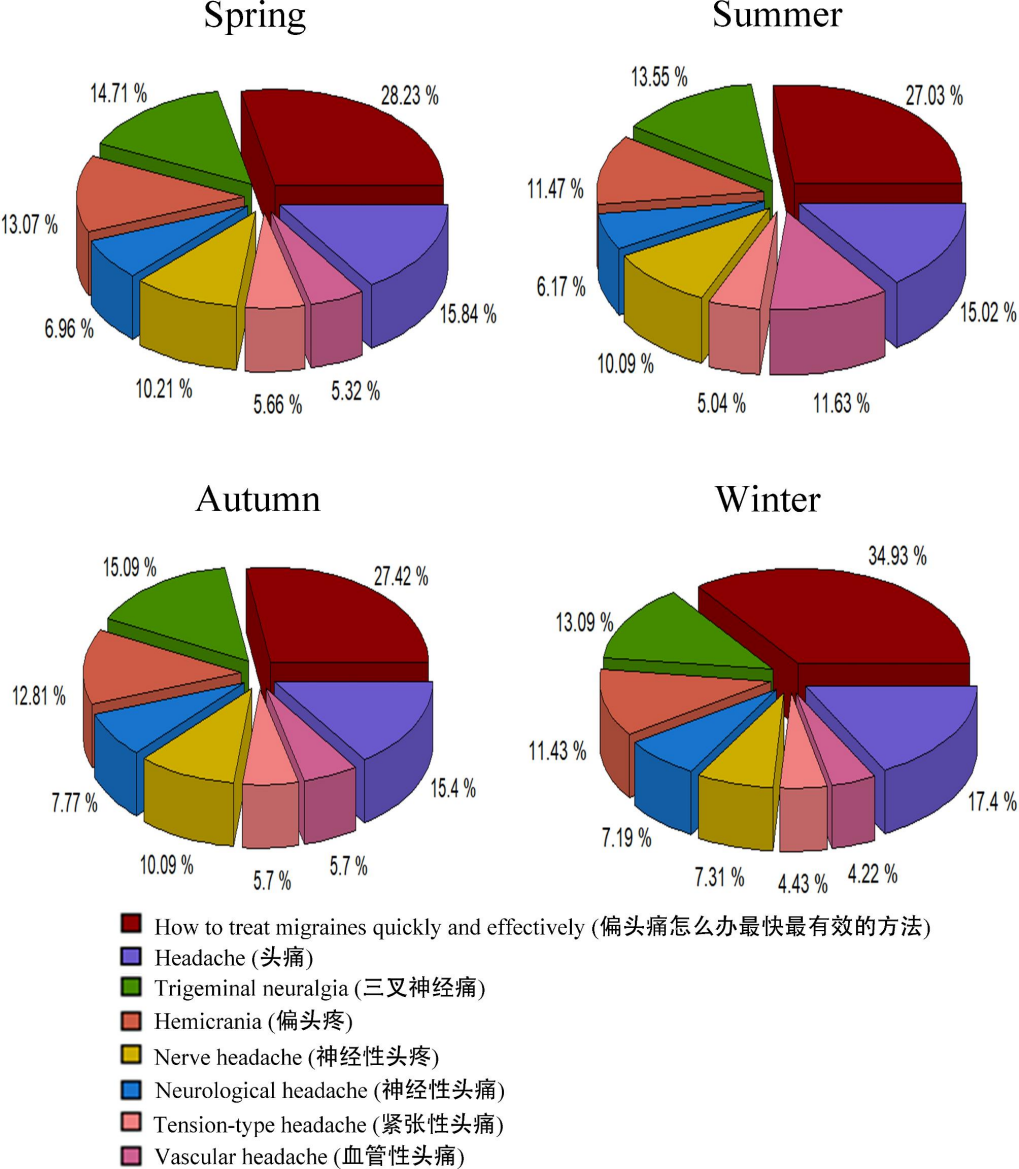
Related demand graphs based on migraine-related Baidu Index data in Mainland China during the year of 2021

Regardless of the season, the most commonly searched term related to migraine was “How to treat migraine quickly and effectively (偏头痛怎么办最快最有效的办法)”. This query consistently ranked at the top position across all seasons, accounting for approximately 30% of the total search demand among the eight terms we examined. Notably, during the winter season, this search term reached its highest proportion, peaking at 34.9% of the total top eight demands (Fig. 4). Following closely in second place was “Headache (头痛)”, which accounted for approximately 15.0-17.4% of the total top eight search demands. “Trigeminal neuralgia (三叉神经痛)” consistently represented a significant portion, ranging from 13.0 to 15.0% across different seasons within the total top eight search demands (Fig. 4).

## Discussion

In this study, we used BI data to investigate the spatiotemporal distribution of migraine in Mainland China and identified search trends, seasonal patterns and regional distribution variations according to migraine. The findings of a strong positive correlation between the BI data and prevalence estimates from the Global Burden of Disease study for migraine suggest that the Internet search behavior of Baidu users in China can serve as a reliable source for monitoring the prevalence of migraine.

The observed gradual increase in migraine-related BI over the years in our study aligns with previous research findings, confirming a growing trend in migraine prevalence in China [28]. These trends are further substantiated by the GBD 2019 data, which reveals an escalating prevalence of migraine in recent years, particularly in the East Asia region, with an average annual percent change (AAPC) of approximately 23%, far exceeding the global AAPC of 5% [29]. This convergence of evidence underscores the mounting significance of migraine as a prevalent health concern in China and East Asia. This may be attributed to several factors, such as changes in lifestyle and environmental factors [30]. Among these factors, excessive use of electronic devices has been identified as a significant risk factor, with an odds ratio of 1.21, for the development of migraine and various other types of headaches, as reported in a study conducted among adolescents [31]. In addition to digital screen-related factors, stress-induced poor sleep quality assumes a significant role in the development of migraine [32]. Moreover, emerging evidence suggests that ambient air pollution such as fine particulate matter (PM2.5) and volatile organic compounds (VOCs), along with climate change also play pivotal roles in the context of migraine and its evolving epidemiology [33]. Furthermore, rising rates of smoking initiation, and elevated stress levels have all been reported as risk factors for migraine [30, 34]. These factors may be closely intertwined with the evolving lifestyle patterns and socio-economic dynamics in China, further highlighting their potential role in the occurrence of migraine [35].

Our study revealed a distinct seasonal pattern in the search behavior for migraine across Mainland China. The peak period nationwide was consistently observed during the months of March, April, and May each year, although varying degrees of seasonal strength were observed across different regions. These results are in line with former researches demonstrating a seasonal variation in migraine prevalence, with the highest attach rates reported in spring [36–38]. However, another study conducted in northern Norway reported the peak of migraine attacks occurring during summer [39]. This discrepancy could be supported by the finding that elevated relative humidity during warm seasons serves as an independent risk factor for migraine [33]. While it can be challenging to identify a specific time of the year deemed worse than others for migraine worldwide, the consistent finding of spring as the peak season across four different regions suggests that spring may require more intervention for migraine in Mainland China.

Regional disparities of migraine-related BI were observed in this study. The combination analyses based various geographical methodology showed that most migraine cases concentrated along the eastern coastal area with the middle and lower Yangtze River area identified as the hotspot, followed by a gradual decrease from the east towards the west and northwest. The findings of this study align with another investigation conducted in 2019, which revealed a prevalence rate of reaching 33% for headache disorders (including migraine and tension-type headache) in the eastern region, in contrast to the western region, where the prevalence rate remained notably lower, at less than 29% [35]. Several factors, including socioeconomic status, environmental factors, and genetic factors may contribute to the regional distribution of migraine prevalence [40–42]. The significant developmental disparity between the eastern and western regions, coupled with a larger population, likely accounts for the notable concentration of migraine in the eastern region [35]. Additionally, unhealthy lifestyle choices, dietary habits, and high levels of stress may further contribute to the prevalence of migraine in East China [43, 44]. It is noteworthy that Jiangxi Province, located in the eastern region, was identified as a significant Low-High Outlier, indicating a need for further investigation into the factors contributing to its outlier status. Possible factors, such as comparatively underdeveloped economy compared to the surrounding regions, should be considered in exploring the underlying reasons.

The most commonly searched term related to migraines was “how to treat migraines quickly and effectively (偏头痛怎么办最快最有效的办法)” which not only signifies the quest for symptom relief but also underscores the inclination of migraine sufferers towards self-treatment. This further substantiates the importance of disseminating extensive medical knowledge on migraines and the necessity of providing comprehensive information on treatment options, highlighting the need for heightened awareness and education regarding appropriate management strategies for migraines.

While our study highlights the growing concern regarding migraine in China, it is essential to acknowledge that migraine remains a significantly underdiagnosed, undertreated, and under-prioritized condition within the country’s healthcare delivery systems. According to data from the China Health Insurance Research Association (CHIRA) database, only 26.4% of patients with migraine received prescriptions for acute medication, and preventive medications were administered to a mere 15% of patients [45]. Alarmingly, approximately 40% of individuals with migraine in China have never consulted a physician, as reported in a comprehensive review [28]. In China, while efforts have been made to enhance the continuing education of general practitioners regarding migraine, the tendency of migraine patients toward self-management is prevalent [28, 46]. Consequently, there is an urgent need for public education initiatives targeting migraine to ensure that individuals with this condition receive accurate information and appropriate guidance for effective management. This is particularly crucial, especially during the winter months when the demand for self-help strategies reaches its peak, and during the spring when the search for migraine is at its highest. Furthermore, considering the regional disparities in migraine prevalence, targeted policy interventions of health care resources allocation should be taken into consideration.

This study adds to the growing body of literature on the use of Internet search data to explore spatiotemporal patterns of health conditions. The Internet has emerged as a valuable source of information for medical-related queries, particularly for those who experience mild or moderate symptoms and tend to self-medicate [47]. Findings from this study are consistent with the GBD data and previous studies, suggesting that Baidu Index data is a reliable proxy for migraine prevalence in Mainland China. Besides, this study not only reveals the efficacy of using Baidu Index for studying health conditions but also provide a promising and efficient avenue for future research in other areas. By leveraging the power of Internet search data, researchers can explore various aspects of health and gain valuable insights for informed decision-making.

Several limitations should be noted when interpreting the results. First, our study relies on Internet search data, which may not capture the entire population’s search behavior, particularly among those who do not use the internet or those who do not search for medical-related queries in Baidu. Second, our study only focuses on the search term “migraine (偏头痛)”, which may not capture all the different types of headache disorders. Third, our study only covers a 9-year period, which may not capture long-term trends in migraine prevalence. However, it is important to note that the Baidu search engine has a penetration rate of over 90% in China, which increases the credibility and representativeness of our study results. Moreover, the backend processing of keywords by Baidu enhances the accuracy of the data and the ability to capture meaningful patterns. It’s important to mention that our study did not conduct gender and age-related analyses due to the absence of a mandatory real-name system for Baidu users, and analyses based on such data could introduce potential bias. In this study, population standardization was intentionally omitted. Caution should be taken when interpreting our findings because our primary focus was on studying the inherent spatiotemporal patterns of migraine cases, rather than delving into the factors contributing to these patterns. Further studies are necessary to validate our findings and explore additional factors contributing to the temporal and spatial distribution of migraine.

## Conclusion

Findings from this study indicate a growing concern regarding migraine in China, reflected in the consistent upward trend in migraine-related BI data. Notably, the spring season, particularly March to May, stands out with consistently high search volumes related to migraine. This seasonal pattern underscores the need for focused public health initiatives and resource allocation during this period. Geographically, the majority of migraine cases are concentrated in eastern China, particularly along the middle and lower reaches of the Yangtze River. These regional clusters emphasize the importance of tailored healthcare planning. To address the rising trend of migraine cases, there is a pressing need for a concentrated allocation of medical resources, particularly during the spring months. Furthermore, prioritizing the dissemination of migraine-related health information and guidance is crucial.

### Electronic supplementary material

Below is the link to the electronic supplementary material.

Supplementary Material 1

## Data Availability

The GBD data are available at https://vizhub.healthdata.org/gbd-results/. The Baidu Index data can be obtained at https://index.baidu.com/v2/index.html#/. All the data used in this study can be obtained from the corresponding authors upon reasonable request.

## Abbreviations

BI: Baidu index
GBD: Global Burden of Disease study
STL: Seasonal-Trend decomposition procedure based on Loess
YLDs: Years lived with disability
IT: Information technology
