# Supplementary material for: Spatiotemporal distribution of migraine in China: analyses based on baidu index
Source: BMC Public Health. 2023 Oct 10;23:1958. doi: 10.1186/s12889-023-16909-9 (PMC10563210; doi:10.1186/s12889-023-16909-9)
Supplement: Supplementary file 1 — Supplementary Material 1 [file 12889_2023_16909_MOESM1_ESM.docx]

Supplemental materials for

**Spatiotemporal distribution of migraine in China: analyses based on Baidu Index**

Liling Lin, Mengyi Zhu, Junxiong Qiu, Qiang Li, Junmeng Zheng, Yanni Fu, Jianwei Lin

**This file includes:**

**Figure S1.** Normality test for the remainder of the Seasonal-Trend decomposition procedure based on Loess method.

**Figure S2.** Ordinary Kriging method of migraine-related BI data in Mainland China.


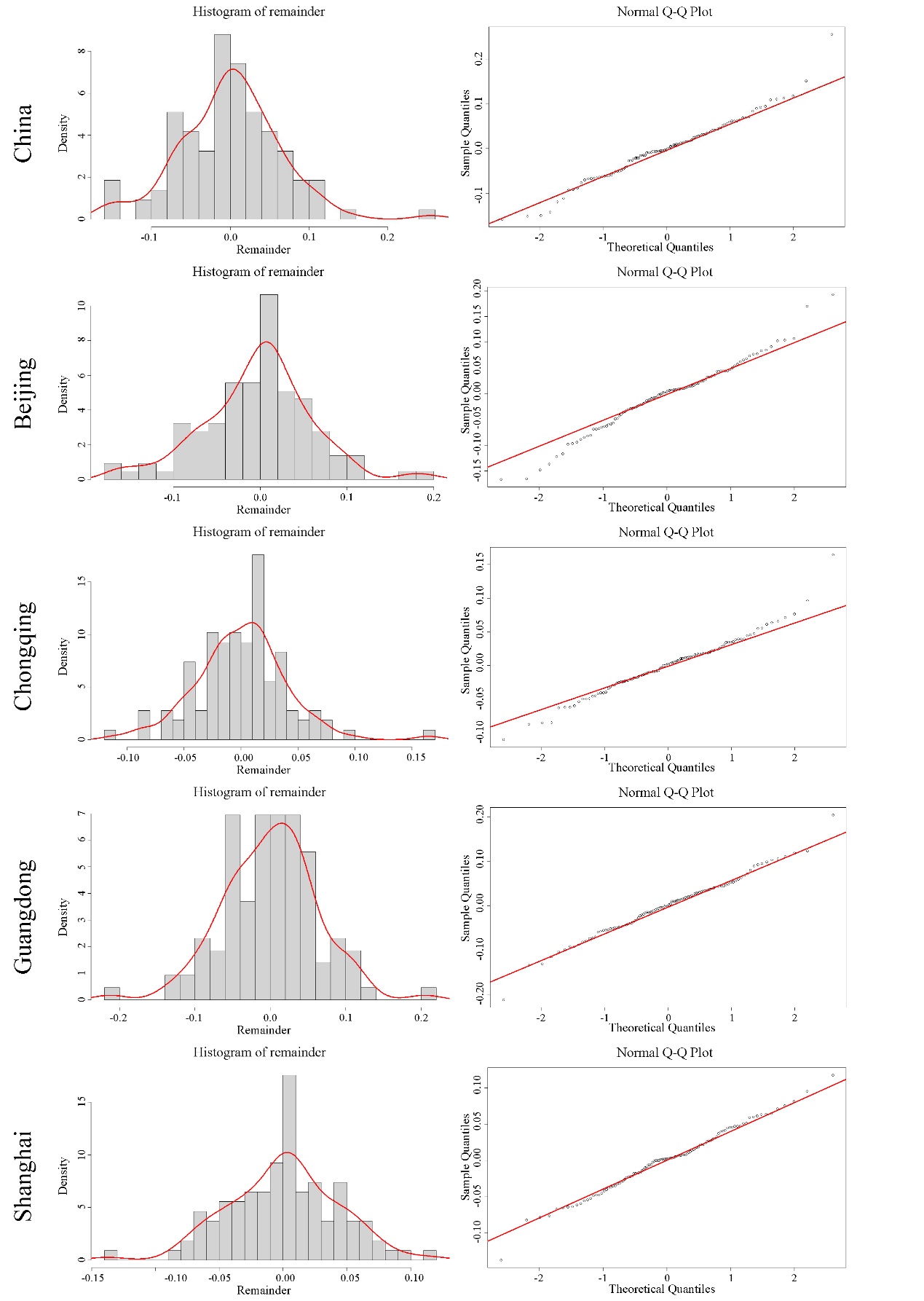


**Figure S1.** Normality test for the remainder of the Seasonal-Trend decomposition procedure based on Loess method.


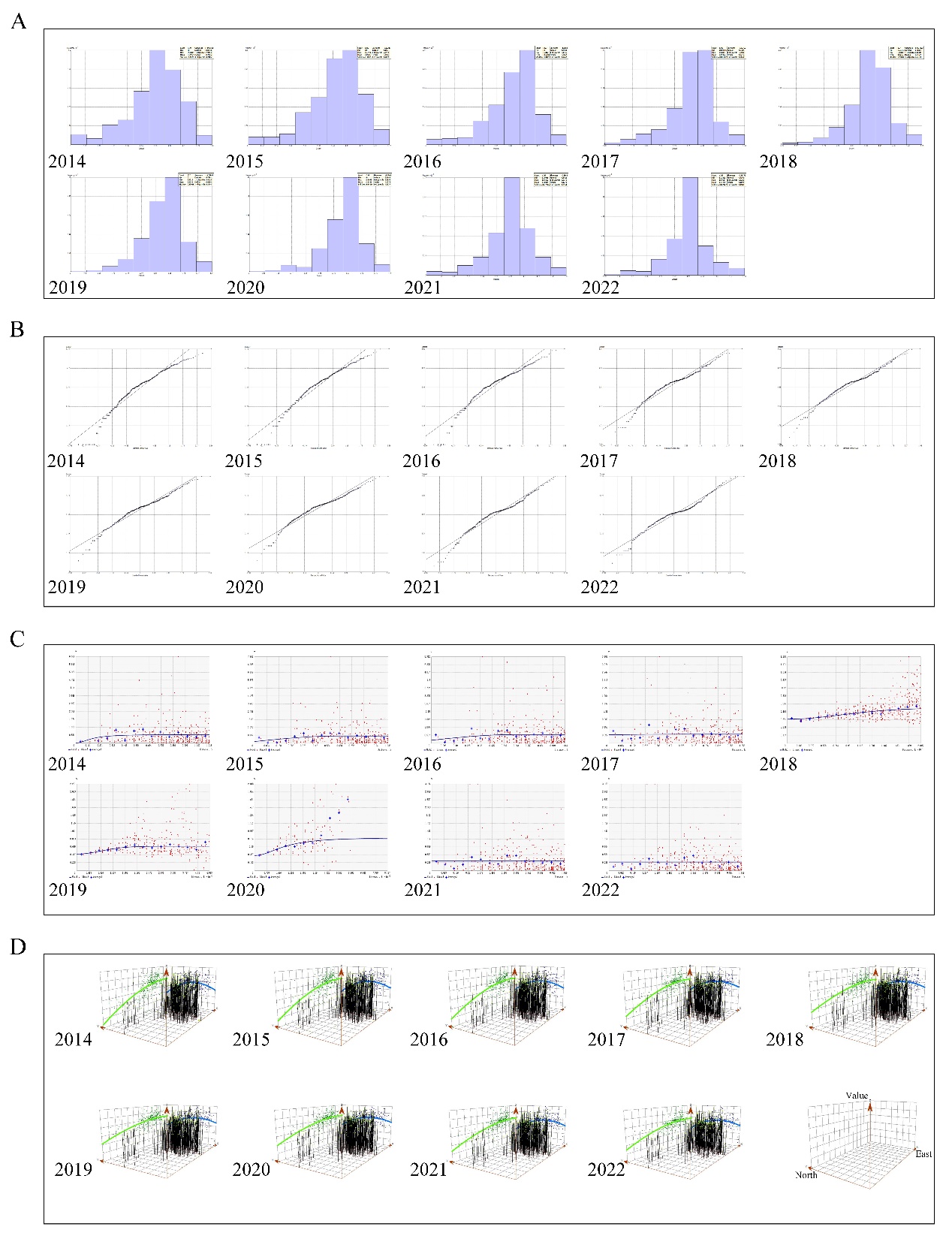


**Figure S2.** Ordinary Kriging method of migraine-related BI data in Mainland China. Data were log-transformed before analyses. **(A)** Histograms of BI in mainland China; **(B)** Normal QQ Plot of BI in mainland China; **(C)** Trend analysis of BI in mainland China; **(D)** Semivariogram of BI in mainland China. BI, Baidu Index.
